# Cellular FRET-Biosensors to Detect Membrane Targeting Inhibitors of N-Myristoylated Proteins

**DOI:** 10.1371/journal.pone.0066425

**Published:** 2013-06-18

**Authors:** Arafath Kaja Najumudeen, Monika Köhnke, Maja Šolman, Kirill Alexandrov, Daniel Abankwa

**Affiliations:** 1 Turku Centre for Biotechnology, Åbo Akademi University, Turku, Finland; 2 The University of Queensland, Institute for Molecular Bioscience, Brisbane, Queensland, Australia; Stanford University, United States of America

## Abstract

Hundreds of eukaryotic signaling proteins require myristoylation to functionally associate with intracellular membranes. N-myristoyl transferases (NMT) responsible for this modification are established drug targets in cancer and infectious diseases. Here we describe NANOMS (NANOclustering and Myristoylation Sensors), biosensors that exploit the FRET resulting from plasma membrane nanoclustering of myristoylated membrane targeting sequences of Gα_i2_, Yes- or Src-kinases fused to fluorescent proteins. When expressed in mammalian cells, NANOMS report on loss of membrane anchorage due to chemical or genetic inhibition of myristoylation e.g. by blocking NMT and methionine-aminopeptidase (Met-AP). We used Yes-NANOMS to assess inhibitors of NMT and a cherry-picked compound library of putative Met-AP inhibitors. Thus we successfully confirmed the activity of DDD85646 and fumagillin in our cellular assay. The developed assay is unique in its ability to identify modulators of signaling protein nanoclustering, and is amenable to high throughput screening for chemical or genetic inhibitors of functional membrane anchorage of myristoylated proteins in mammalian cells.

## Introduction

Covalent protein lipidation is an important protein modification in eukaryotic cells that enables the reversible association of hundreds of proteins with the membrane. Protein lipid transferases, i.e. prenyl-transferases, myristoyl- and palmitoyl-transferases attach lipid moieties in particular to signaling proteins [1]. Most of these transferases are well established drug targets in a number of diseases, most notably cancer [2]–[5]. They may be regarded as surrogate targets, as their protein substrates such as for instance Ras-superfamily proteins are very difficult to target directly.

Inhibition of lipid transferases renders their protein substrates cytoplasmic thereby dramatically reducing their biological activity as exemplified by the important oncoproteins Src- [6], [7] and Ras [8], [9]. It has been shown that ∼40% of membrane associated Ras molecules are concentrated in 6–20 nm signaling packages, termed nanoclusters that contain 6–8 Ras molecules [10]–[12]. Nanoclustering is essential for Ras activity and disruption of clustering leads to a reduction in Ras activity and prevents its robust biological signaling [13]. These experimental data are supported by computational simulations, which suggest that lipid-anchors of Ras spontaneously organize into membrane nanocluster in mammalian cells [14], [15]. Due to the high local protein density, nanoclustering can be detected by FRET, if the nanoclustered polypeptides are fused to FRET-fluorophores, such as mCFP and mCit [16]–[19].

While there are already numerous inhibitors for the Ras modifying farnesyltransferase and geranylgeranyltransferase in preclinical and clinical trials [20], [5], there is a paucity of potent and specific inhibitors of other lipid transferases, including N-myristoyltransferases (NMT). N-myristoylation is the co-translational and irreversible attachment of a myristoyl-group to an N-terminal glycine (typically in the consensus sequence MGXXXS/T) via an amide linkage [21]–[23]. It involves N-terminal methionine cleavage by one of the two human methionine amino-peptidases (Met-AP 1 and 2), followed by NMT catalyzed transfer of myristate from myristoyl-CoA to the glycine on position two. Bioinformatic analysis suggests that 0.5% of the eukaryotic proteome is myristoylated making this one of the most frequent posttranslational protein modifications [24], [25]. In vertebrates two N-myristoyltransferase homologues NMT 1 and 2 have been identified, but only limited information is available on their peptide substrate specificity [26]–[28]. Heterotrimieric G protein alpha-subunits of the Gi-subfamily are co-translationally myristoylated on their N-terminus and undergo cycles of re/depalmitoylation in cells that regulate their membrane localization [29], [30]. Similarly, Src-family kinases are targeted to the plasma membrane by myristoylation in combination with palmitoylation or a polybasic stretch of amino acids at their N-terminus [1].

NMT1 (but not NMT2) knockdown was shown to inhibit tumor growth, which can be rationalized by the fact that NMT substrates include proto-oncogenic Src-family kinases [31], [32]. This validates NMT as a direct target in cancer [32]. In addition, myristoylated small GTPases of the Arf-family and NMT itself have been confirmed as targets in human pathogenic parasitic diseases caused by *Trypanosoma brucei* and *Leishmania major* [24], [33]. Finally, NMT is also a pharmacological target in viral and bacterial infections, as viruses and bacteria hijack the myristoylation machinery of the host cell [34].

Currently only few inhibitors of membrane anchorage of myristoylated proteins are known. Inhibitors of Met-AP, such as pyridine-2-carboxylic acid derivatives are known to specifically block human Met-AP1 and prevent progression through G_2_/M of the cell cycle [35]. Inhibitors of Met-AP2, such as fumagillin and derivatives, inhibit angiogenesis, while dual specificity bengamide A affects the cell cycle [36], [37].

First generation NMT-inhibitors such as Tris dibenzylidenacetone dipalladium (Tris DBA) inhibited NMT1 with IC_50_ = 1.0 µM *in vitro*, blocked MAPK and Akt signaling in cells, and demonstrated anti-tumor activity in a mouse melanoma model [38]. Despite this high medical relevance, only recently a first nanomolar inhibitor of *Trypanosoma brucei* NMT, the pathogen of sleeping sickness, was identified [39].

The myristoyl group is often complemented by palmitoylation for plasma membrane targeting [1]. Palmitoylation is carried out by the DHHC-family of palmitoyltransferases (PATs) [40], [41]. The most commonly used inhibitor of protein palmitoylation is 2-bromopalmitate [1], [42]. However, this compound is active only at relatively high concentrations of 100 µM as a broad-spectrum inhibitor that also affects myristoylation. Other identified lipidic inhibitors were shown to exhibit only low mM activity [43]. However, recent insight into the palmitoylation cycle of the cell has led to the development of promising inhibitors of acyl-protein thioesterase 1 (APT 1), which hydrolyzes the palmitoyl-ester bond [44], [45].

Here we report the design and application of three FRET-biosensors that can detect membrane anchorage of N-myristoylated proteins in mammalian cells. These biosensors exploit nanoclustering-induced FRET making them therefore in addition uniquely suitable for the detection of novel nanocluster modulators. Such modulators may represent a novel class of pharmacological compounds that attenuate the action of membrane anchored signaling molecules. We demonstrate that these biosensors report on the inhibition of NMTs and Met-APs and can potentially be employed in cell-based high-throughput screening.

## Results and Discussion

### Design and Application of NANOclustering and Myristolyation Sensors (NANOMS)

In order to design biosensors that would detect functional membrane anchorage of myristoylated proteins in mammalian cells, we exploited the fact that fluorescently tagged myristoylated membrane anchors can display high FRET due to nanoclustering [16].

**Figure 1:**
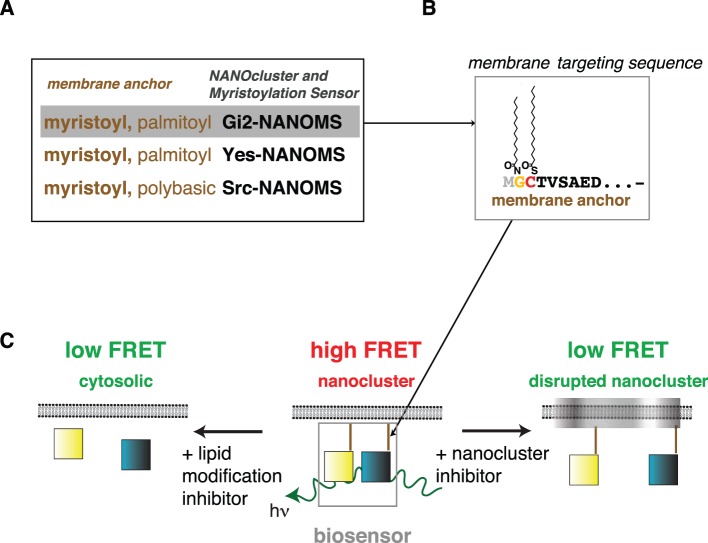
Design and reporting principle of NANOMS. FRET-biosensor design of the three different NANOMS. (**A**) The myristoylated N-terminal membrane-targeting motifs of mouse Gα_i2_ (residues 1–35), human Yes (1–17)- and human Src (1–16)-kinases were genetically fused to the N-terminus of fluorescent proteins mCFP or mCit. The sequence of the employed membrane-targeting motifs can be found in **Table S2**. (**B**) Intracellular processing involves cleavage of the N-terminal methionine (grey) by methionine amino-peptidase (Met-AP), NMT-mediated myristoylation on glycine 2 (yellow) and depending on the motif cysteine-palmitoylation (red). (**C**) Lipid modified reporters spontaneously organize into plasma membrane nanocluster. Tight packing of membrane targeted donor (mCFP)- and acceptor (mCit)-fluorophores (blue and yellow squares, respectively) in nanocluster leads to FRET. FRET can decrease due to loss of nanoclustering or cytoplasmic redistribution of the NANOMS after inhibitor treatment. As membrane anchorage is required for the functioning of myristoylated proteins, NANOMS report on functional membrane anchorage.

We constructed three myristoylation biosensors that exploit nanoclustering-FRET, by genetically fusing the N-terminal membrane targeting sequences of the heterotrimeric G protein subunit Gα_i2_, Yes- or Src-kinases to the fluorescent proteins mCFP and mCit (**Figure 1**). We termed the resulting FRET-biosensors NANOMS (NANOclustering and Myristoylation Sensors).

On a two dimensional surface, such as a biological membrane, FRET depends on the donor-acceptor ratio and the density of the fluorophores [46]. We therefore analyzed the dependence of FRET on the acceptor expression level at constant donor-acceptor ratio of 1∶1, using a flow cytometer with a previously established protocol [16], [17], [19]. This allowed us to monitor the full expression range of the biosensors in cells at high throughput (tens of cells per second). At high acceptor expression levels, we determined the FRET-parameter E_max_ (**Figure S1**), which reports on membrane nanoclustering and therefore also on functional membrane anchorage (a prerequisite for nanoclustering) [16], [17], [19] (**Figure 1**). As compared to imaging-based high-content data analysis, this parameter elegantly integrates the intricate subcellular distribution of the fluorescently tagged membrane-targeting motifs into a single, relevant FRET-parameter.

### NANOMS Report on Chemical Inhibition of NMT in Mammalian Cells

**Figure 2:**
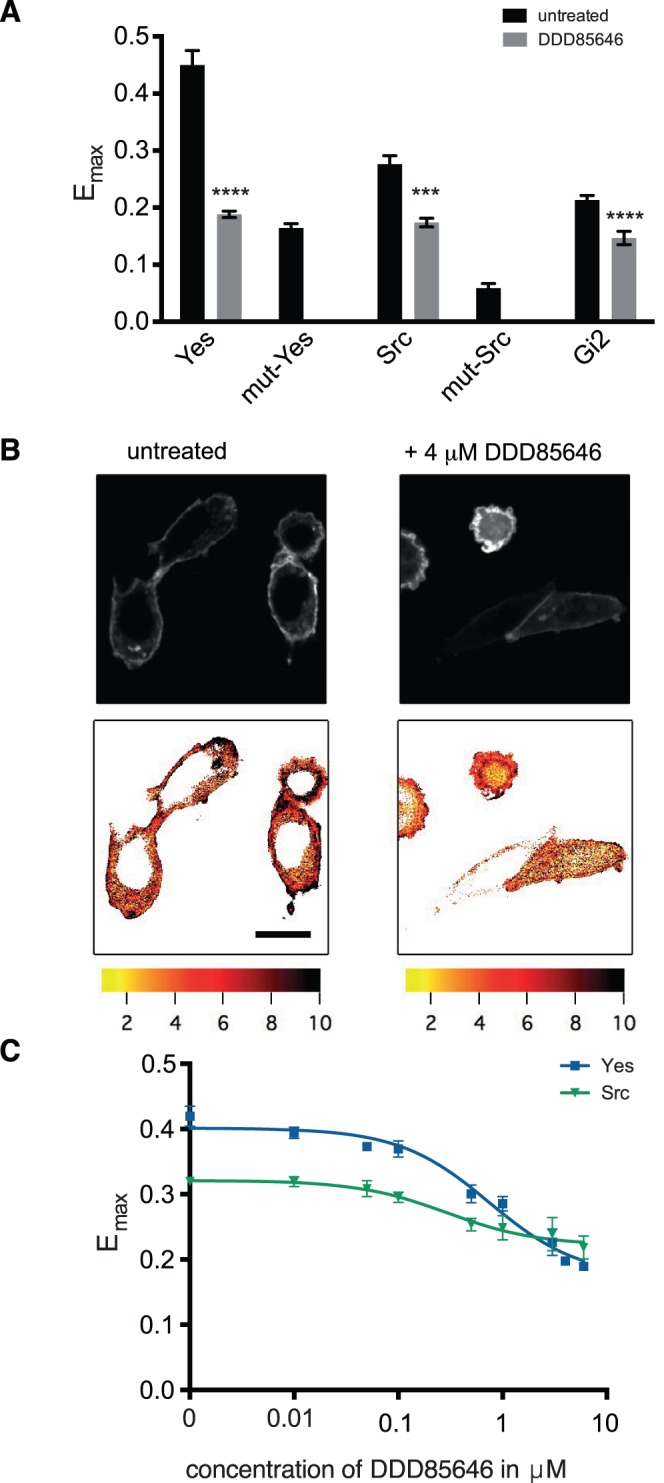
NANOMS report on chemical inhibition of NMT. (**A**) FRET-responses of Yes-, Src- and Gi2-NANOMS transfected BHK cells treated with 4 µM of the specific NMT inhibitor DDD85646. The error bars denote the s.e.m (n = 5). Samples were statistically compared with the untreated control. See Methods for more information on statistical analysis. (**B**) Confocal sensitized acceptor FRET-imaging of Yes-NANOMS expressed in BHK cells. Cells were treated as indicated. Top row shows acceptor channel images, and bottom row FRET images. The look-up table shows the FRET-index FR, color coded with high FRET levels in black and yellow (value 1) indicating no FRET. Scale bar is representative for all images and corresponds to 10 µm. (**C**) Dose-response curves of the effect of DDD85646 on the E_max_ values of Yes- and Src-NANOMS expressed in BHK cells (n = 6).

BHK cells expressing Yes-NANOMS showed a very high E_max_ value of >0.4, indicating strong nanoclustering. When they were treated with 4 µM of the potent NMT-inhibitor DDD85646 [39], the E_max_ value significantly decreased by >50% to ∼0.2. FRET-imaging confirmed the loss of FRET on the plasma membrane, due to cytoplasmic redistribution of the biosensor (**Figure 2B**). The E_max_-level found after DDD85646-treatment corresponded to the FRET level of a non-myristoylatable mutant of this biosensor, where the myristoylated glycine was converted into an alanine (**Figure 2A**).

The other biosensors showed a much lower E_max_ values of ∼0.3 (Src-NANOMS) and ∼0.2 (Gi2-NANOMS), which were also significantly reduced by DDD85646 treatment. However, FRET-levels were not quite as low as that of the G/A-myristoylation site mutated Src-biosensor, suggesting that the compound did not fully inhibit nanoclustering-FRET of the Src-biosensor with our treatment protocol (**Figure 2A**). Both Yes- and Src-NANOMS dose-dependently responded to inhibition of NMT-activity by DDD85646-treatment, with similar submicromolar IC_50_ values of 0.75±0.15 µM and 0.30±0.06 µM, respectively (**Figure 2** **C**).

We confirmed the response to NMT-inhibition in HEK293 EBNA cells, where we observed that the nanoclustering FRET parameter E_max_ of Yes- and Src-NANOMS was significantly reduced upon DDD85646-treatment (**Figure S2**).

Using Gi2-NANOMS in BHK cells, we demonstrated that the response to inhibitors of myristoylation was specific, as other lipid modification inhibitors such as a farnesyl transferase inhibitor (FTI) and a statin did not elicit a response (**Figure S3A**). Testing of weaker inhibitors of NMT, such as myristoleic acid and TDP (Tris (dibenzylideneacetone) dipalladium), or halogenated-palmitates, as *bona fide* inhibitors of palmitoylation, (**Figures S2 B,C, S3 B–D**) correspondingly resulted in no or only small decreases of the E_max_ values of the biosensors in both BHK and HEK293 cell lines. Differences between observed cellular and the reported *in vitro* activity, may reflect limited bioavailability of these compounds.

In summary, NANOMS respond specifically to potent inhibitors of myristoylation. The high E_max_ and dose-response characteristics of Yes-NANOMS suggest that it is well suited for monitoring myristoylation inhibition in cells.

### NANOMS Report on siRNA-mediated NMT Knockdown

**Figure 3:**
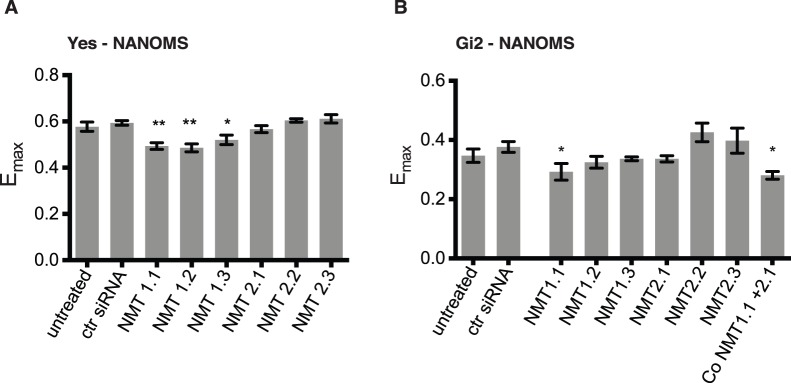
NANOMS reports on RNAi-mediated depletion of NMT. (**A**) HEK293 EBNA cells transiently expressing Yes-NANOMS and (**B**) HEK293 cells transiently expressing Gi2-NANOMS were treated with NMT1 or NMT2 specific siRNAs or control siRNA. Knock-down efficiencies are shown in **Figure S4**. The characteristic E_max_-value was determined on flow cytometric FRET data. The error bars denote the s.e.m (n = 4). Samples were statistically compared with the untreated control. See Methods for more on statistical analysis.

To further confirm that NANOMS report on NMT activity in mammalian cells, we knocked down human NMT1 and NMT2 in HEK293 cell lines and monitored the effect on the FRET of Yes- and Gi2-NANOMS. In agreement with our chemical inhibition data, knockdown of NMT1 lead to a significant decrease in E_max_ for both biosensors (**Figure 3A, B**), while knockdown of NMT2 alone did not lead to any response. Consistent with the latter observation, co-knockdown of NMT1 and NMT2 in cells expressing Gi2-NANOMS did not augment the response as compared to NMT1-inhibition alone (**Figure 3B**). This indicates that NMT1 is the principal modifying enzyme for both Yes- and Gi2-NANOMS. Therefore our knockdown experiments confirmed that Yes- and Gi2-NANOMS specifically report on the NMT-activity in cells.

### Screening of a Cherry-picked Chemical Library with Yes-NANOMS

**Figure 4:**
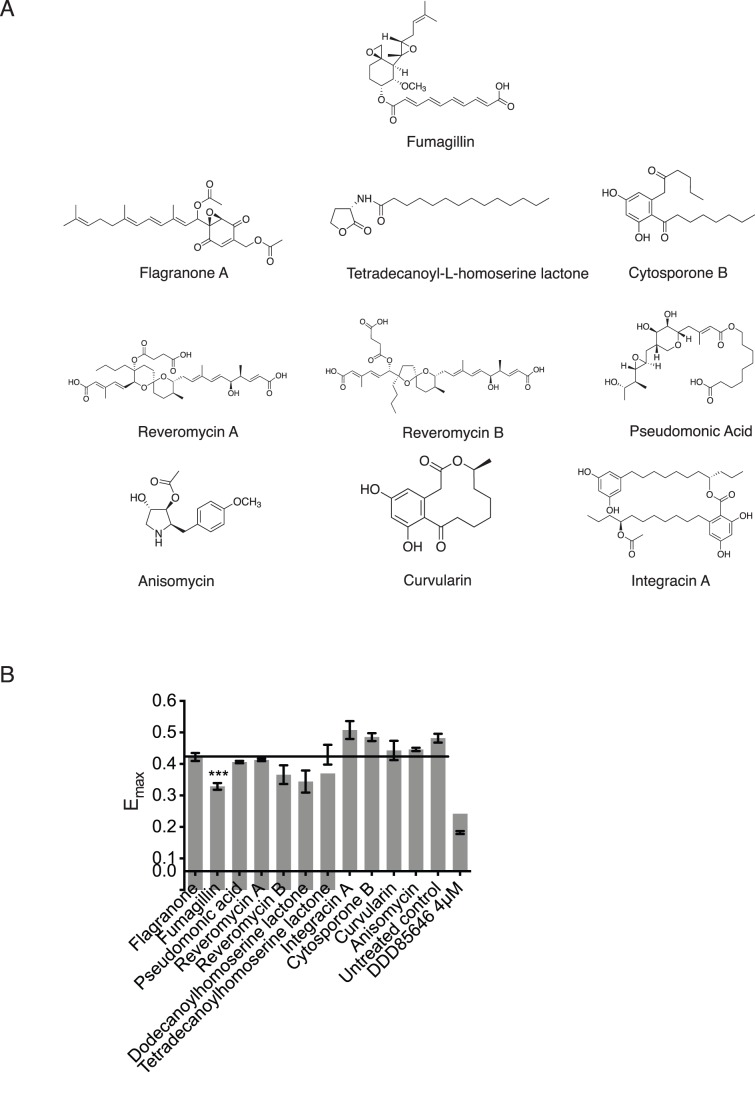
Cherry-picked chemical compound library screen with Yes-NANOMS. (**A**) Chemical structures of chemical compounds that were included in the cherry-picked chemical library. (**B**) BHK21 cells were transfected with Yes-NANOMS and screened with shown chemical compounds at a final concentration of 10 µM/mL. FRET-response of Yes-NANOMS to the chemical compounds is represented with E_max_ values. Block line indicates the average E_max_ and the error bars denote the s.e.m (n≥4). Samples were statistically compared with the untreated control. See Methods for more on statistical analysis.

Finally we explored whether Yes-NANOMS is suitable for screening of chemical compounds that would block its membrane attachment. Inhibitors of Met-APs have been successfully used to block membrane anchorage and activity of myristoylated proteins [36]. Fumagillin is a known inhibitor of human methionine amino-peptidase 2 (Met-AP2) that possesses anti-angiogenic activity [37]. Another new class of dual-specific Met-AP inhibitors are bengamides and derivatives, which efficiently block Src-activity and have significant antitumor activity *in vivo* [36], [47], [48]. We therefore collected compounds with chemical structures similar to fumagillin (oxygenated six-membered ring) or bengamide A (long chain fatty acids) and tested their activity on BHK cells expressing Yes-NANOMS (Figure 4A).

The assay had an excellent Z′-score of 0.60, when using the compound DDD85646 at 4 µM as a positive control. Two homoserine lactones and reveromycin B appeared to reduce the E_max_ value. However, only fumagillin decreased the E_max_ value highly significantly which is in agreement with its inhibitory activity against Met-AP2 (Figure 4B).

In conclusion, Yes-NANOPS is suitable for screening of chemical compound libraries and should have similar potential also for genetic screening applications.

In summary, our cytometric assay merges the benefits of imaging-based high content screening and plate reader based cellular assays. The E_max_ value rapidly integrates essential features of the subcellular localization that is commonly obtained by cell imaging. On the other hand, the assay can be carried out at a rate comparable to that of conventional plate reader based assays.

Most importantly, our assay has the unique potential for the discovery of nanoclustering modulators of myristoylated proteins, which may provide a new approach for their pharmacological modulation. The importance of nanoclustering has been demonstrated for Ras signaling [17], [49], [50] and by analogy [51], we expect that inhibition of nanoclustering of myristoylated proteins will critically affect their signaling activity, too. Our previous data showed that heterotrimeric G protein alpha subunits from the Gα_q_ and Gα_i/o_ subfamily laterally segregate into distinct membrane nanodomains [16], [24], [33]. This may suggest that with the help of our FRET-biosensors inhibitors against specific nanoclusters can be developed.

Our assay is flexible and can be adapted to other cell lines, provided that they allow for sufficiently high expression of the biosensor to determine the E_max_ parameter. It is even conceivable to implement the biosensors in protozoan pathogens, e.g. in order to understand the mechanism of action of membrane organization disrupting compounds [34], [52].

These features, the discovery of novel nanocluster inhibitors and the potential for a cellular high-throughput assay, clearly distinguish our assay from existing formats. The standard assay for N-myristoylation is radioactive and albeit successful even in the high-throughput setting [38], [39], not really optimal towards that goal. Only recently two complementary non-radioactive *in vitro* assays have been published. The first detects fluorometrically the released CoA-SH and is thus generally sensitive to hydrolyzing compounds in the screening context [39], [53]. In the second assay a click-chemistry amenable myristate-analogue is utilized and detected by an ELISA-assay like procedure in both cellular and tissue samples [54].

In conclusion, the assays described here have a unique potential for the discovery and validation of both chemical and biological modulators of functional membrane anchorage of myristoylated proteins in mammalian cells.

## Methods

### Plasmids and Molecular Cloning

Plasmids pN_Src16_mCit-N1, pN_Src16_mCFP-N1, pN_mutSrc16_mCit-N1, pN_mutSrc16_mCFP-N1, pN_Yes17_mCit-N1, pN_Yes17_mCFP-N1, pN_mut Yes17_mCit-N1 and pN_mutYes17_mCFP-N1 were cloned in a two-step PCR reaction where the specific N-terminal membrane-targeting sequences were added to the N termini of mCit or mCFP from the vector pmCit-N1 or pmCFP-N1. In the case of plasmids pN_Src16_mCit-N1 and pN_Src16_mCFP-N1, 16 amino acids from N-terminus of Homo sapiens c-Src (NM 005417) were added using forward primer 5′-CCCAAGGATGCCAGCCAGCGGCGCCGCGTGAGCAAGGGCGAG-3′ (Sigma Aldrich) in the first PCR reaction and forward primer 5′-CGCTAGCACCATGGGTAGCAACAAGAGCAAGCCCAAGGATG-3′ in the second PCR reaction. To construct plasmids pN_mutSrc16_mCit-N1 and pN_mutSrc16_mCFP-N1 in which the myristoylated glycine 2 was mutated to alanine, forward primer in the second PCR reaction was: 5′-CGCTAGCACCATGGCTAGCAACAAGAGCAAGCCCAAGGATG-3′. In the case of plasmids pN_Yes17_mCit-N1 and pN_Yes17_mCFP-N1, 17 amino acids from the N-terminus of Yamaguchi sarcoma viral oncogene homolog 1 (NM 005433) were added using forward primer 5′- GAAAACAAAAGTCCAGCCATTAAATACAGAGTGAGCAAGGGCGAGGAG-3′ in the first PCR reaction and forward primer 5′-CGCTAGCACCA TGGGCTGCATTAAAAGTAAAGAAAACAAAAGTC-3′ in the second PCR reaction. To construct plasmids pN_mutYes17_mCit-N1 and pN_mutYes17_mCFP-N1 where the myristoylation site, glycine 2, was mutated to alanine and palmitoylation site cysteine 3, was mutated to serine, forward primer in the second PCR reaction was: 5′- CGCTAGCACCATGGCCAGCATTAAAAGTAAAGAAAACAAAAGTC-3′. Reverse primer used in all PCR reactions was: 5′- CGCGGCCGCTTTACTTGTACAGCTCGTCCATG-3′. PCR products were gel purified and subcloned into pCRII-Blunt-TOPO (Life Technologies Corporation) and from there they were subcloned into the BsrGI and NheI restriction sites of the pmCFP-C1 vector. The backbone of pmCit-N1 and pmCFP-N1 vectors is a pEGFP-N1 vector (Clontech Laboratories, Inc.) in which EGFP was replaced by mCit or mCFP between NheI and BsrGI restriction sites. The plasmids pN_Gi2.mCFP-N1 and pN_Gi2.mCit-N1 have been described previously [16]. Final constructs were verified by sequencing (GATC Biotech AG, Cologne, Germany).

### Cell Culture

BHK 21 cells (Sigma-Aldrich) were cultured in Dulbecco’s modified Eagle’s medium (DMEM, Invitrogen) supplemented with 10% fetal bovine serum (FBS) (Invitrogen, # 26140079), 100 U/ml penicillin G and 100 U/ml streptomycin (Invitrogen, # 15070-063). HEK293 cells (Sigma-Aldrich) were cultured in DMEM containing 10% FBS, 1% Glutamine (Invitrogen, # 25030-081), 1% non-essential amino acids (Invitrogen, # 11140050), 100 U/ml penicillin G and 100 U/ml streptomycin. HEK293 EBNA cells were cultured adherent in Dulbecco’s modified Eagle’s medium (DMEM, Sigma-Aldrich, # D6171), containing 5% FBS, 100 U/mL penicillin (Sigma-Aldrich), 100 µg/mL streptomycin (Sigma-Aldrich), L-glutamine (Sigma-Aldrich, # G7513). All cells were incubated at 37°C with 5% CO_2_. Transfections were performed with jetPRIME (Polyplus transfection) according to the manufacturer’s instructions in a 6-well plate. On the next day cells were transferred to a 96-well plate with a density of 5×10^4^ cells per well. The compound stocks were dissolved in DMSO (Sigma Aldrich, # 41641) and diluted in cell–culture medium for experiments to give a final DMSO concentration below 0.3%. All inhibitors (**Table S3**) were added to cultured cells 24 h after transfection and thereafter cultures were incubated for an additional 24 h.

### Confocal FRET-imaging

BHK 21 cells were grown on coverslips, transfected with FuGene6 (Roche), after 24 hours they were treated with compounds and 24 hours later fixed with 4% PFA (Sigma, # P6148) in PBS and mounted on microscopic slides using Mowiol 4–88 (Sigma, # 81381). A Zeiss LSM 510 confocal microscope with a 63×/1.4 oil DIC immersion objective was used to record 12 bit 512×512 fluorescent images, using 200 µm pinhole size and 0.09 µm pixel size in the frame mode with 8× averaging. Sensitized acceptor emission FRET images were acquired using the three-cube method, with the following settings of donor (ex 405 nm, 5% laser power, em 480–520 nm), acceptor (ex 514 nm, 2% laser power, em 535–590 nm) and FRET (ex 405 nm, 5% laser power, em 535–590 nm) channels. Lsm images were converted into Tiff using Fiji [55] and processed further to calculate the sensitized acceptor FRET-index FR [56] in a custom written procedure in IgorPro6 (Wavemetrics, Oregon), as described previously [17], [57].

### Chemical Screen

Chemical library screens were performed with BHK21 cells seeded in 6-well plates and transfected with Yes-NANOMS using jetPRIME (Polyplus transfection) following the manufacturer’s instructions. 18 h after transfection cells were split to clear flat bottom 96 well plates with 5×10^4^ cells per well with complete DMEM medium. After the cells have attached (usually 5–7 h), they were treated with compounds from the library. The compound stocks were prepared in 100% DMSO and stored at −20°C until use. Prior to use the compounds were brought to room temperature and were diluted to a final drug concentration of 10 µM/mL in the growth medium with a final DMSO concentration of under 0.3%. The cells were treated with the compounds for 24 h; afterwards cells were detached with 75 µL of 10 mM EDTA in PBS and fixed using an equal volume of 4% PFA (Sigma, # P6148) in PBS for 15 minutes at room temperature. The samples were stored at 4°C until analysed. The screen was performed with at least three independent biological repeats. Each 96-well plate was designed to have an internal DDD85646 dose response control.

### Flow Cytometric FRET Analysis

The measurements were performed on a FACS LSRII (BD biosciences) equipped with a high throughput sampler, using the following filters for donor- (405 nm excitation, 450/50 nm emission filter), acceptor- (488 nm, excitation, 585/42 nm emission filter) and FRET-channel (488 nm excitation, 530/30 nm emission filter). The flow cytometer data were analyzed for FRET with a custom written procedure in IgorPro6 (Wavemetrics), as described [16], [19]. In brief, doublet discrimination was implemented to measure signals of single cells. For normalized acceptor level calibration, cA, FITC beads (Bangs Laboratories) with a defined size and fluorescein content were used as described previously. A mCFP-mCit fusion protein was used to calibrate for the FRET efficiency and donor-acceptor ratio. Only cells with a donor mole-fraction, x_D_ = 0.5±0.1 were analyzed. The E_max_ value was determined as described [17].

### siRNA Knockdown Experiments and RT-PCR Quantification

HEK293 were seeded in a 12-well plate and siRNA transfection was performed on the next day with Lipofectamine RNAiMax (Invitrogen) according to the manufacturer’s instructions with a final concentration of 40 nM siRNA in the medium. Cells were harvested 48 h after transfection; RNA extraction and cDNA synthesis (CellSure, Bioline) were performed according to the manufacturer’s protocol. The real-time PCR reactions consisted of cDNA template (diluted 1∶20), forward and reverse primers (200 nM final concentration), and Platinum SYBR Green qPCR Supermix-UDG (Invitrogen) in a total volume of 20 µL. Glyceraldehyde 3-phosphate dehydrogenase (GAPDH) was used as normalization reference. Quantitative real time PCR was carried out in triplicate on indicated number of independent templates on a 7500 Real-Time PCR System (Applied Biosystems). For analysis of the Ct values the ΔΔCt method was applied. To test for contamination standard control PCR reactions were performed. For FACS analysis of the knockdown, DNA transfection of the biosensor constructs with FuGene6 was performed 24 h after siRNA transfection. The cells were analyzed for FRET on the flow cytometer as described above, 48 h after siRNA transfection. A table with employed siRNAs is given in **Table S1**.

### Statistical Analysis

Significant differences between mean values of inhibitor treated samples and mean values of untreated samples were analyzed using two-tailed Student’s t-tests in GraphPad Prism. Confidence p-levels are given above columns and in addition indicated by asterisks, with * denoting p<0.05, ** denoting p<0.01, and *** denoting p<0.001. The mean IC_50_ values for inhibition were calculated from six independent experiments and data were analyzed in GraphPad Prism by nonlinear regression analysis on log (inhibitor) versus (normalized) response with a Hill Slope of −1.0 using the Marquardt method. Z′ scores were calculated on the control data in the chemical screen from the following formula Z′ = 1– (3⋅σ_pos_ +3⋅σ_neg_)/(|µ_pos_ – µ_neg_|), with σ_pos/neg:_ SDs of positive and negative controls, respectively, and µ_pos/neg_: averages of positive and negative controls, respectively [58].

## Supporting Information

Figure S1 Plots of processed cytometer FRET-data of Yes-NANOMS with treatment. Three sets of examples for the cytometer FRET-curves from the Yes-biosensor with different levels of DDD85646 mediated NMT inhibition are shown. BHK cells transiently overexpressing the biosensor were treated with increasing concentrations of DDD85646 as indicated. The data processing is done as described in methods. The plots show the dependence of the FRET-efficiency, E, on the accessible acceptor concentration at a constant donor-acceptor ratio of ∼ 1∶1. The characteristic E_max_-value was determined by exponential fitting (red curve) of single cell data (black dots).
(EPS)

Figure S2 Response to myristoylation inhibitors in HEK293 EBNA cells. FRET-responses of Yes- and Src-NANOMS transfected HEK293 EBNA cells treated with the specified concentrations of (**A**) DDD85646, (**B**) myristoleic acid (MA) and (**C**) Tris (dibenzylideneacetone) dipalladium (TDP). The Emax-value was determined on flow cytometric FRET data. The error bars denote the s.e.m. Samples were statistically compared with the untreated control. See Methods for more information on statistical analysis.
(EPS)

Figure S3 Response to weak myristoylation and palmitoylation inhibitors in BHK cells. (**A**) Control experiment showing that chemical inhibition with a farnesyl-transferase inhibitor or HMG-CoA inhibitor did not lead to a significant response. Gi2-NANOMS transfected BHK cells were treated with the specific farnesyltransferase inhibitor FTI277 (a CAAX-box peptidomimetic) or the statin compactin (5 µM). The effect on the characteristic E_max_-value was determined by flow cytometric FRET analysis. (**B**) Yes- and Src-NANOMS transfected BHK21 cells did not show a significant response to other weak myristoylation inhibitors like Tris (dibenzylideneacetone) dipalladium (TDP) (5 µg/mL) or myristoleic acid (MA) (0.2 mM). (**C**) FRET-responses of Gi2-NANOMS transfected BHK cells treated with indicated concentrations of myristoleic acid. A significant reduction of FRET is seen only at concentrations above 1 mM. For comparison, in a radioactive *in vitro* assay with human NMT the IC_50_ of myristoleic acid was 0.85 µM [1]. (**D**) FRET-responses of Gi2-NANOMS transfected BHK cells treated with 100 µM of the weak acylation inhibitors 2-bromopalmitate and 2-fluoropalmitate with 5 µM compactin as a negative control. Of note, fatty acid derivatives are known to affect both palmitoylation and myristoylation [1]–[3]. We previously confirmed this by observing that myristoleic acid dose dependently decreased the E_max_ of our biosensor Ras-NANOPS [4]. Therefore, we cannot rule out that the observed response of Gi2-NANOMS to 2-fluoropalmitate reflects inhibition of NMTs. The characteristic E_max_-value was determined on flow cytometric FRET data. The error bars denote the s.e.m. Samples were statistically compared with the untreated control. See Methods for more information on statistical analysis.
(EPS)

Figure S4 Knock-down efficiencies of NMT1 and NMT2. (**A, B**) RT-PCR data of siRNA mediated NMT knockdown. The knockdown efficiencies of (**B**) NMT1 and (**C**) NMT 2 transcripts were determined by quantitative real-time PCR. HEK293 cells were treated with three different NMT1 or NMT2 siRNAs or control siRNA (final concentration 40 nM). The mRNA expression levels were normalized to GAPDH expression levels and are expressed relative to untreated control. Mean values and SEM of three repeats are given. Samples were statistically compared with siRNA control. See Methods for more information on statistical analysis.
(EPS)

Table S1 Sequences of siRNA oligonucleotides used in this study.
(DOC)

Table S2 Membrane-targeting peptide sequences used to design the respective NANOMS in this study.
(DOC)

Table S3 Chemical compounds used in the study.
(DOC)
