# Correction: Cellular FRET-Biosensors to Detect Membrane Targeting Inhibitors of N-Myristoylated Proteins

**DOI:** 10.1371/annotation/98f18cba-53d7-43a5-9f23-4c35d0fd4ce1

**Published:** 2014-01-03

**Authors:** Arafath Kaja Najumudeen, Monika Köhnke, Maja Šolman, Kirill Alexandrov, Daniel Abankwa

The first seven bars in the bar graph of Figure 4 were erroneously altered. Please see the corrected Figure 4 here: http://plosone.org/corrections/pone.0066425.g004.cn.tif

