# Supplementary material for: Cellular FRET-Biosensors to Detect Membrane Targeting Inhibitors of N-Myristoylated Proteins
Source: PLoS One. 2013 Jun 18;8(6):e66425. doi: 10.1371/journal.pone.0066425 (PMC3688908; doi:10.1371/journal.pone.0066425)
Supplement: Table S1 — Sequences of siRNA oligonucleotides used in this study. (DOC) [file pone.0066425.s005.doc]

**Table S1:** Sequences of siRNA oligonucleotides used in this study.

| NMT1 1 | AAA UCU GUC ACC UCU CCG UUU GCG U |
| --- | --- |
| NMT1 2 | AGA AUA AGC AGC UUU GAG ACU CUU G |
| NMT1 3 | AGA AUA AGC AGC UUU GAG ACU CUU G |
|  |  |
| NMT2 1 | GGA UUU AUG ACA GUG UGA AGA AGA U |
| NMT2 2 | UAU GGG CUU AGG AAG AAC CAC UCC C |
| NMT2 3 | CCG GGA GCA CAU UAU UGA CAC GUU U |
|  |  |
