# Supplementary material for: Cellular FRET-Biosensors to Detect Membrane Targeting Inhibitors of N-Myristoylated Proteins
Source: PLoS One. 2013 Jun 18;8(6):e66425. doi: 10.1371/journal.pone.0066425 (PMC3688908; doi:10.1371/journal.pone.0066425)
Supplement: Table S2 — Membrane-targeting peptide sequences used to design the respective NANOMS in this study. (DOC) [file pone.0066425.s006.doc]

**Table S2:** Membrane-targeting peptide sequences used to design the respective NANOMS in this study.

| **NANOMS** | **peptide sequence derived from parent proteins as described in Methods** |
| --- | --- |
| Gi2 | MGCTVSAEDKAAAERSKMIDKNLR EDGEKAAREVK |
| Yes | MGCIKSKENKSPAIKYR |
| Src | MGSNKSKPKDASQRRR |
