# Supplementary material for: Cellular FRET-Biosensors to Detect Membrane Targeting Inhibitors of N-Myristoylated Proteins
Source: PLoS One. 2013 Jun 18;8(6):e66425. doi: 10.1371/journal.pone.0066425 (PMC3688908; doi:10.1371/journal.pone.0066425)
Supplement: Table S3 — Chemical compounds used in the study. (DOC) [file pone.0066425.s007.doc]

**Table S3: Chemical compounds used in the study**

| **Inhibitor** | **IC_50_ –**  **in vitro** | **IC_50_ –**  **cells** | **Provider** | **References** |
| --- | --- | --- | --- | --- |
| Myristoleic Acid | 0.3 μM |  | Sigma Aldrich | Pasha et al. 2004,  Selvakumar et al.  2007 |
| Tris (dibenzylideneacetone) dipalladium (TDP) | 1.0 μM |  | Santa Cruz | Bhandarkar et al., 2008 |
| FTI-277 |  | 0.3 μM | Calbiochem | Lerner et al., 1995 |
| 2-bromopalmitate |  | 100 μM | Sigma Aldrich | Webb et al. 2000 |
| 2-fluoropalmitate |  | 100 μM | United Bioresearch | Kohnke et al., 2012 |
